# Laparoscopic Management of Blunt Pancreatic Trauma in Adults and Pediatric Patients: A Systematic Review

**DOI:** 10.1155/2023/9296570

**Published:** 2023-09-28

**Authors:** Barbara Catellani, Daniela Caracciolo, Paolo Magistri, Cristiano Guidetti, Nunzia Menduni, Helen Yu, Roberta Odorizzi, Gian Piero Guerrini, Roberto Ballarin, Stefano Di Sandro, Fabrizio Di Benedetto

**Affiliations:** Hepato-Pancreato-Biliary Surgery and Liver Transplantation Unit, University of Modena and Reggio Emilia, 41124 Modena, Italy

## Abstract

**Background:**

Pancreatic trauma is an uncommon injury that occurs usually in a young population and is frequently overlooked and not readily appreciated on initial examination. Nowadays, the diagnosis and management of pancreatic trauma are still controversial, and there is no gold standard for the treatment. The aim of this study is to describe our experience in the management of blunt pancreatic trauma with a laparoscopic approach and review the literature on laparoscopic management of pancreatic trauma.

**Methods:**

A systematic literature review was performed, and 40 cases were reported and analysed; 10 cases were excluded because the complete data were not retrievable. We also reported our experience with the case of an 18-year-old male diagnosed with a deep laceration of the pancreas between body and tail, involving the main pancreatic duct, and with a concomitant hematoma. The patient underwent exploratory laparoscopy with abdominal toilet, necrosectomy, and suture of main pancreatic duct; the total blood loss was less than 200 ml, and the total operative time was 180 minutes. The patient recovered uneventfully and was discharged on the 6th postoperative day.

**Results:**

30 patients with pancreatic trauma, 10 adults and 20 pediatrics (mean age 28.2 years and 10.5 years), underwent a total laparoscopic approach: 2 distal pancreatic-splenectomy, 22 spleen-preserving distal pancreatectomy, and 6 laparoscopic drainage. The mean operative time for the adult and pediatric populations was 160.6 and 214.5 minutes, the mean estimated blood loss was 400 ml and 75 ml, and the mean hospital stay was 14.9 and 9 days, respectively.

**Conclusion:**

Laparoscopic management for pancreatic trauma can be considered feasible and safe when performed by an experienced laparoscopic pancreatic team, and in such a setting, it can be considered a viable alternative to open surgery, offering the well-known benefits of minimally invasive surgery.

## 1. Background

Pancreatic trauma is a relatively rare injury that occurs in 0.2-3.1% of patients with blunt trauma and in 1-12% of patients with penetrating trauma [1–3]. It is often associated with other intra-abdominal and/or extra-abdominal injuries (50-98% of the cases) [2, 4]. The incidence of injuries to organs other than the pancreas after blunt abdominal trauma ranges from 45% to 85%, while after penetrating trauma, this rate is nearly 100% [3]. Isolated pancreatic injuries are rare, occurring in only 0.7% of all abdominal traumas [2]. Furthermore, traumatic pancreatic injuries are associated with high morbidity and mortality rates, respectively, ranging between 30-40% and 9-34%; however, these rates are primarily related to injuries of other associated organs [1, 3, 5, 6].

Pancreatic trauma is associated with considerably high morbidity and mortality in cases of delayed diagnosis, incorrect classification of the injury, or delays in treatment [7]. The mortality rate directly attributed to pancreatic injuries ranges from 2% to 17% and further increases with delayed diagnosis [1, 3, 8]. Therefore, the aim must be an early diagnosis and an appropriate treatment.

Patients with concomitant solid organ injuries, especially hepatobiliary or duodenal lacerations, are most commonly treated surgically [9]. However, there is still no consensus over the optimal management of pancreatic trauma [3], and several issues regarding the surgical and nonsurgical management of pancreatic trauma are still controversial. In the pediatric population, higher grades of pancreatic injury and overall injury severity are strongly associated with the use of operative pancreatic management, while pancreatic head injuries are associated with nonoperative pancreatic management [10].

The traditional approach for major pancreatic trauma is open exploratory laparotomy, but recently, laparoscopic surgery has been applied in this context by analogy with pancreatectomies for other conditions. A systematic literature review has been conducted to define the role of the laparoscopic approach in those cases in terms of safety and feasibility.

## 2. Methods: Review Process

### 2.1. Study Design

Our review was designed according to the Preferred Reporting Items for Systematic Reviews and Meta-Analyses (PRISMA) statement, while the authors predetermined the eligibility criteria for the study. Two investigators (BC and DC) independently searched the literature. All discrepancies during the data collection, synthesis, and analysis were resolved by consensus of the two authors (PM and CG). All retrospective clinical studies, case reports, and review that concern minimally invasive approach to pancreatic trauma were included in the present review. Late laparoscopic treatments for posttraumatic pseudocyst were excluded.

### 2.2. Literature Search

**Figure 1:**
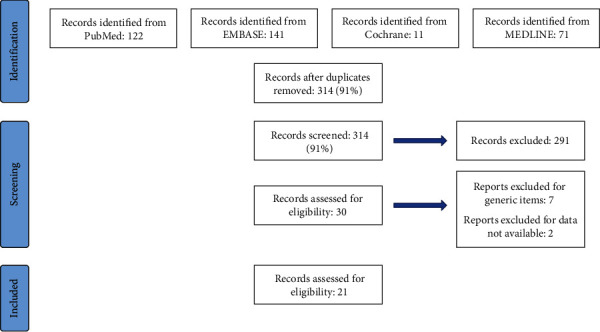
Review diagram.

We systematically searched the literature using the PubMed, MEDLINE, Embase, and Cochrane library databases for articles published from January 2000 until December 2022. Our search included the words “laparoscopic management AND pancreatic trauma”, “pancreatic trauma AND laparoscopy”, “minimally invasive approach AND pancreatic trauma”, and “laparoscopy AND blunt pancreatic trauma”. Only papers in English language were considered eligible for inclusion; 3 articles not in English were excluded (one of them was written in Cyrillic and two in German). Our search strategy disclosed 314 publications, of which thirty were full papers on laparoscopic management for pancreatic trauma. Twenty-three full papers were examined; however, two studies were not included in the analysis because data were not available. Finally, 21 articles for a total of 30 patients were included in the review. The flow diagram in Figure 1 shows the search process.

### 2.3. Data Collection

For each case, we considered the following variables: age, gender, type of trauma, clinical manifestation at presentation, laboratory tests, diagnostic technique, grade of pancreatic trauma according to the American Association for the Surgery of Trauma grading system (AAST) (Table 1) [10], associated injuries, time interval for surgery, type of laparoscopic management (drainage, suture repair, or resection), operative time, blood loss, complications, mortality, hospital stay, readmission with 30 days, and follow-up. The categorical variables were described with frequency and percentages, and the continuous variables were expressed as the mean ± standard deviation.

We also reported our experience with the management of a blunt pancreatic trauma with a laparoscopic approach and analysed the literature about laparoscopic management of pancreatic trauma.

### 2.4. Outcomes

Our primary outcome was management strategy, in particular the feasibility and safety of laparoscopic approach. Secondary outcomes were mortality and major complications.

## 3. Results

### 3.1. Laparoscopic Approach for Pancreatic Trauma

Forty cases of pancreatic trauma managed by mini-invasive laparoscopic approach were reported in the literature during the study period (Figure 1). Among these, ten cases were excluded because no data could be retrieved. Finally, 30 patients were included in the review process. Patients' characteristics and demographic data are listed in Tables 2 and 3; 10 adult patients (over 18 years), with a mean age of 28.2 years (±5.5, range 18-34), 7 females and 3 males, and 20 pediatric patients (under 18 years), with a mean age of 10.5 years (±4.17, range 3-17), 7 females and 13 males, were reported.

### 3.2. Causes and Presentation of Pancreatic Trauma

Approximately 77.9% of pancreatic injuries in adults (7/9) were caused by vehicle crashes as a result of impact with the steering wheel, motorbike, or bicycle handlebars; other causes were being struck in the abdomen by an opening gate, a horse kick, and a stab wound to the lower back. In one case, the trauma description was not available. In the pediatric population, approximately 50% of pancreatic injuries (5/10) were caused by bicycle handlebars or dirt bike; in two cases, by impact to the abdomen during sports; in three cases, by a car accident (auto vs. pedestrian); and in the other ten cases, the trauma description was not available. The accident mechanism and bruising are important indicators of the nature of the injuries.

Abdominal pain with increased amylase and/or lipase with or without tenderness and vomiting are the most frequent clinical manifestations. However, an increased amylase level in the serum is unreliable for the diagnosis since it occurs in 85% of cases but can only be expected 3 h after an accident at the earliest [17]. In children, elevation of serum amylase is unreliable as well, being predictive in only 49% of cases [27]. At the presentation, all patients were hemodynamically stable and underwent diagnostic CT scan and/or MRI; in the 2 patients, the correct diagnosis was obtained only with the MRI and in 1 case with endoscopic retrograde cholangiopancreatography (ERCP). Classification of severity of pancreatic injury is according to the pancreatic injury scale described by the American Association for the Surgery of Trauma (AAST) and distinguishing pediatric from adult population: 1 grade II lesion (5%), 17 grade III lesion (85%), and 2 grade IV lesion (10%) in children, and 2 grade II lesion (22.25%), 5 grade III lesion (55.5%), and 2 grade IV lesion (22.25%) are reported in adults; in one case, the grade of pancreatic trauma was not available (pancreatic laceration). Moreover, in adults, pancreatic trauma was associated with other intra-abdominal injuries in 6 cases (37.5%): grade I liver lacerations/hematoma in 3 cases; grade II splenic laceration in 2 cases (one of these with left pleural effusion); and duodenal hematoma in 1 case. In children, there were reported 4 cases of other intra-abdominal injuries (20%): one case of grade II duodenal injury with duodenal hematoma and a grade II hepatic injury; 2 cases grade II splenic laceration with one case of left pleural effusion; and one case of splenic hematoma.

### 3.3. Management

In the adult population, the mean time interval to surgery was 39 hours (±26.7, range 6-72). All patients underwent a total laparoscopic approach: 1 distal pancreatic-splenectomy (DPS), 4 spleen-preserving distal pancreatectomy (SPDS), one of which was followed 1 day later by intramuscular islet autotransplantation, and 5 laparoscopic drainages (LD), associated with an endoscopic stent and one to a jejunostomy.

The two patients with grade II trauma and the patient with generic pancreatic laceration underwent laparoscopic drainage, and the five patients with grade III trauma underwent spleen-preserving distal pancreatectomy (SPDP) within 28.5 hours (±29.9, range 6-72). The two patients with grade IV trauma underwent laparoscopic drainage in one case with jejunostomy within 72 hours and in the other case with an endoscopic stent within 48 hours.

The mean operative time was 160.6 minutes (±27.3, range 122-180), with a mean estimated blood loss of 400 ml (±122.5, range 200-500) and a mean hospital stay of 14.9 days (±12.7, range 4-44). The overall complications rate was 40%, and according to the Clavien-Dindo grading system, they were all Clavien grade I-II. The morbidity included pancreatic fistula (grade A or B) in 3 cases (one of these with pancreatic collection); pancreatic collection in 2 cases. No readmissions within 30 days were observed, and no fatal event occurred. At follow-up, only one case of asymptomatic stricture of the pancreatic duct was reported.

In the pediatric population, the mean time interval to surgery was 34.2 hours (±18.2, range 23-72), and in 3 cases, surgery was performed in an emergency setting. The patient with grade II trauma associated with grade II duodenal injury and grade II hepatic injury was treated with conservative management, but after seven days, he underwent laparoscopic drainage with gastrojejunostomy. It was complicated by duodenal bleeding and luminal bleeding and treated with an emergency open Whipple pancreaticoduodenectomy. The seventeen patients with grade III trauma underwent SPDP; the two patients with grade IV trauma underwent one DPS and one SPDP. All of them underwent by laparoscopic approach. The mean operative time was 214.5 minutes (±92.4, range 146-344), with a mean estimated blood loss of 75 ml (±25, range 50-100) and a mean hospital stay of 9 days (±4.6, range 3-20). The complications rate was 40% (8/20): in 7 cases (35%) Clavien grade I-II, in one case (5%) Clavien grade III-IV. The morbidity included one duodenal intraluminal bleeding, two pancreatic fistula (grade A), one postoperative ileus, one wound infection, one pancreatitis, and an abdominal wall hematoma in one case. In this population, two cases of readmissions within 30 days were observed; in one case, for a percutaneous drainage of the collection; in the second case, data were not available; the mortality rate was 0%.

### 3.4. Case Report

**Figure 2:**
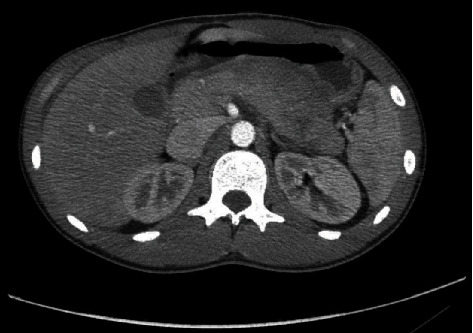
CT scan at presentation in emergency room.

**Figure 3:**
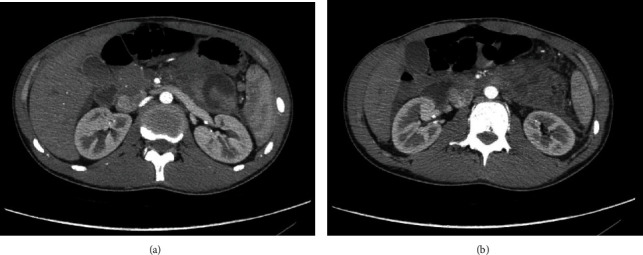
CT scan on the 2nd day posttrauma. (a) Peripancreatic blood collection (3.5 cm). (b) Increase of fluid collection peripancreatic, perisplenic, and along the left lateral conal fascia.

**Figure 4:**
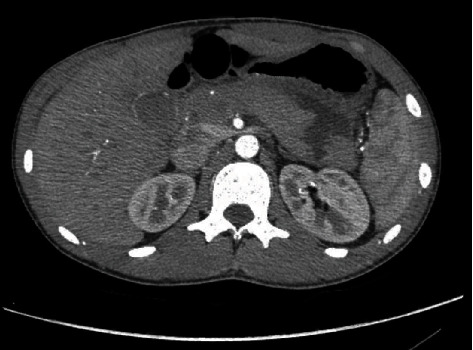
CT scan on the 6th day posttrauma: laceration of the pancreas between the body and tail.

An 18-year-old male presented to the emergency room of a peripheral hospital for a blunt abdominal trauma that occurred during a football game (a blow with the elbow). On the examination, he was hemodynamically stable, conscious, and oriented, with vomiting, sweating, pain, and tenderness over the epigastrium, extending to the left hypochondrium. He did not show signs of peritonism and/or retroperitoneal haemorrhage. The focused assessment with sonography (FAST) was positive for perihepatic, peripancreatic, and pelvic fluid collection, without signs of parenchymal lesions. Blood tests showed increased amylase (986 U/L), lipase (156 U/L), and bilirubin (2.35 mg/dl). Edema of the pancreas, hypodense area between body and tail suspect for laceration, single spot of suspect bleeding from the pancreatic tail, and small liver laceration (less than 1 cm and negative for active bleeding) were demonstrated on CT scan (Figure 2). Initially, the patient was managed conservatively with intravenous fluids, analgesics, and clinical assessment. A new CT scan was performed on the 2nd day after trauma, showing a peripancreatic blood collection (3.5 cm), an increase in the peripancreatic and perisplenic fluid collections, while the bleeding spot from the pancreatic tail was reduced, as shown in Figures 3(a) and 3(b). On the 3rd day, the patient was referred to our tertiary center, and after a clinical and biohumoral evaluation, the nonoperative management was confirmed. On the 6th day after the trauma, a CT scan was performed due to the progressive exacerbation of painful symptoms along with high fever. The imaging revealed a wedge-shaped laceration of the pancreas between the body and tail, involving the main pancreatic duct, and enlargement of the blood collection (Figure 4). According to the AAST grading system, a grade III lesion was defined.

**Figure 5:**
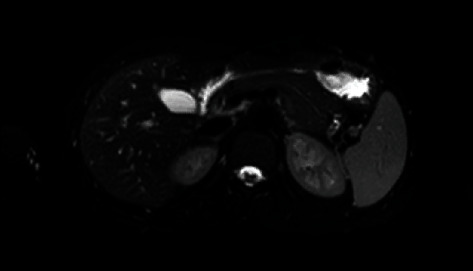
MRI at 24 months of follow-up: no evidence of collections or of pancreatic pseudocysts.

Therefore, an exploratory laparoscopy with abdominal toilet was scheduled for the same day. The patient was placed in a reverse trendelenburg position, and a 10 mmHg pneumoperitoneum was created using a 10-12 mm Hasson's trocar, positioned in the periumbilical region for laparoscopy. Under direct vision, through a 30° optical device, two 5 mm trocars were inserted into the right flank and right pararectal line and one 10 mm trocar into the left pararectal line. A diagnostic laparoscopy was performed without evidence of other solid organ lesions (Supplementary material – video clip (available here)). The gastrocolic ligament was divided through an ultrasonic scalpel, and the lesser sac was entered, revealing significant peripancreatic inflammation, pancreatic contusions, and an organized perisplenic, retrogastric and peripancreatic blood collection extending along the left lateroconal fascia. Intraoperative ultrasound (IUS) was performed to better define the retroperitoneal hematoma surrounding the pancreatic body and to evaluate the splenic inflow that resulted preserved. There was significant peripancreatic fat necrosis without clear anatomic planes. An incision of the collection and necrosectomy were performed. A deep laceration of the pancreas was found, including the division of the main pancreatic duct at the border between body and tail. The IUS confirmed preserved vascularization of the pancreatic tail; therefore, nonresectional management was chosen. One nonabsorbable stitch and two metallic clips were used to selectively suture the proximal pancreatic duct. Conversely, it was not possible to identify the distal pancreatic duct in the tail; therefore, tissue glue and an absorbable fibrin sealant patch were applied. A single surgical drain was put along the pancreatic transection. The total blood loss was less than 200 ml with no need of blood transfusion. The total operative time was 180 minutes. The patient was discharged on the 6th postoperative day, in good conditions and tolerating a diet, with an abdominal drain for the presence of a biochemical pancreatic leak. Although several abdominal US were negative for abdominal collections, amylase could be found in the drained fluid for 1 month (fistula grade B) until the drain was finally removed. The MRI follow-up at 3 months posttrauma was negative for collections or pancreatic pseudocysts. Follow-up at 24 months was as well negative, as shown in Figure 5, and the patient is asymptomatic without endocrine or exocrine deficiency.

## 4. Discussion

Due to its protected retroperitoneal location, lesions of the pancreas are rare, with an incidence between 2 and 12%, and are often misunderstood. The physical signs and symptoms of traumatic pancreatic injury may be nonspecific or even absent and are frequently overlooked and not readily highlighted on initial examination [30]. From the overview of cases analyzed, the population was young, and the clinical presentation on admission was nonspecific with subtle clinical signs; abdominal pain with increased amylase and/or lipase with or without tenderness and vomiting are the most frequent clinical manifestations (14/17, 82.35%). Several studies have observed that a clinical deterioration in traumatic population may, in some instances, be the first clue of an underlying occult or undetected pancreatic injury [11, 28, 30]. Therefore, diagnostic imaging plays an important role in the recognition, evaluation, and follow-up of traumatic pancreatic injuries.

CT is the preferred method for evaluating suspected pancreatic trauma; it could diagnose possible extra-abdominal and/or intra-abdominal injuries, including the staging of pancreatic trauma [4], but in some cases, it is not possible to predict injuries to the DP using CT. According to the literature, in 90% of cases (27/30), a CT scan was diagnostic and defined the grade of pancreatic injury; in only two patients, the correct diagnosis was obtained only with an MRI, and in one case, with an ERCP. Using the AAST classification, the following were identified: 1 grade II lesion (5%), 17 grade III lesion (85%), and 2 grade IV lesion (10%) in the pediatric population, and 2 grade II lesion (22.25%), 5 grade III lesion (55.5%), and 2 grade IV lesion (22.25%) in adults. With this grading system, high-grade injuries are correlated with more serious complications and higher mortality. The Eastern Association for the Surgery of Trauma (EAST) Guidelines recommend nonoperative management for grade I/II pancreatic injuries diagnosed by CT scan and operative management for grade III/IV pancreatic injuries [3].

The management of pancreatic trauma relies on the haemodynamic stability of the patient, the presence of concomitant life-threatening injuries, the location of parenchymal injury, the integrity of the pancreatic duct, the presence of complications like acute necrotising pancreatitis and/or pancreatic fistulae and/or abscess, and the need for damage control procedures [1, 3, 4]. The 3 cases with grade II trauma, after initially conservative treatment, underwent a surgical approach: 2 patients underwent laparoscopic drainage, and one patient, also with grade II duodenal injury and grade II hepatic injury, underwent laparoscopic drainage with gastrojejunostomy after 7 days. The last one was complicated by duodenal bleeding and luminal bleeding and treated with an emergency open Whipple pancreatoduodenectomy. From available data, grades III and IV underwent laparoscopic surgery with a mean time interval of 38.2 hours (±23.2, range 6-72) and in 3 pediatric cases in an emergency. Timing of surgery is crucial for outcomes; once pancreatic duct disruption is identified, surgery should not be delayed because delays in surgical resection can potentially make surgery more difficult in the presence of posttraumatic inflammation (pancreatitis), fibrosis, and/or sepsis [29]. Several authors recommend an early operative intervention to prevent increased morbidity caused by delay [10]. Meier et al. [31] documented benefits in children that underwent pancreatic resection within 72 hours of injury. De Wilt et al. [15] and Nadler et al. [32] showed that a significant morbidity, mortality, and prolonged hospital stay have usually been reported in patients with AAST grade III-V pancreatic injuries being treated conservatively. For major pancreatic injury, an aggressive approach is recommended to reduce the long-term risks of prolonged hospital stay, sepsis, fistula, pancreatic collection, pseudocyst formation, and chronic pancreatitis. The overall complications rate in both populations was 40%; in adults, all cases were Clavien grade I-II, while in children, 7 cases (35%) were Clavien grades I-II and one case (5%), Clavien grades III-IV. No later complications.

In our case, initially a nonoperative management was chosen considering hemodynamic stability and CT imaging. On the first and second days after trauma, the lesion was considered a grade II according to the AAST. Only after worsening of clinical conditions, after 6 days from trauma, was the correct severity grade highlighted by a further CT scan that showed a grade III lesion. The same day, the patient underwent exploratory laparoscopy, and a deep laceration was found and treated with suture of the Wirsung duct and abdominal drainage. A conservative surgical approach was preferred over a distal pancreatectomy to reduce the risk of developing diabetes in the postoperative period given the young age of the patient. The risk of diabetes after trauma is related to the volume reduction of the gland, and it is more frequent after distal pancreatectomy; therefore, islet autotransplantation has been reported as an alternative [12].

Moreover, early detection of disruption of the main pancreatic duct is of paramount importance because such disruption is the main cause of delayed complications like pseudopancreatic cyst and mortality [1, 3, 8]. In the cases analyzed, the mortality rate was 0%, given the early surgery.

Even if there are currently few case reports in the literature on laparoscopic approach for the management of pancreatic trauma, largely due to the rarity of both the condition and the treatment modality, early laparoscopic approach can play a role for the diagnosis and staging of blunt pancreatic injuries and for the management, by providing a valid alternative to the open abdominal surgery [11]. In all reported cases, the laparoscopic approach was used for therapeutic purposes, and in none of the cases represented a bridge to laparotomy, with a 0% conversion rate. Laparoscopy in trauma can be in fact used also as a diagnostic tool to screen for the possible need for laparotomy. This can reduce unnecessary morbidity and mortality and also reduce hospital costs and length of stay [33]. Stringel et al. [34] showed in their work that laparoscopic surgery in pediatric abdominal trauma reduces negative and nontherapeutic laparotomy rates from 60% to 40%, encouraging this approach whenever possible. Other known benefits of minimally invasive surgery are preserved cosmesis and reduced pain [27]. In the literature, the reported complications rate after surgical treatment of pancreatic trauma ranges between 26 and 86% [29], including pancreatic fistula in 8 to 30% [35]; this rate is similar after an open versus laparoscopic approach, but laparoscopy provides a shorter hospital length of stay, a faster recovery, and less pain [35]. Therefore, in selected patients with worsening symptoms, an early laparoscopic approach can be a safe, feasible, and reproducible option and may play a role in the staging and treatment of blunt pancreatic injuries.

It is important to underline that laparoscopic approach in the setting of trauma should only be performed by pancreatic surgeons experienced in laparoscopic surgery due to the increased complexity of these cases, whereby the presence of a large hematoma and increased potential for bleeding frequently result in an unusual surgical anatomy with diminished visualization.

This study has some limits to disclose. For instance, the type of studies included increases the risk of bias from unmeasured confounders, including clinical deterioration and specific radiologic or intraoperative findings; in addition, this study lacks a complete longitudinal follow-up. Further studies will be needed on this topic.

## 5. Conclusion

Laparoscopic management of blunt pancreatic trauma in a hemodynamically stable patient is feasible and safe when performed by an experienced laparoscopic pancreatic surgical teams. In such a setting can be considered as a viable alternative to open surgery, potentially offering the usual benefits of minimally invasive surgery, such as faster recovery, lower morbidity, and respect of the integrity of the abdominal wall in both young and elderly patients.

## Figures and Tables

**Table 1:** The American Association for the Surgery of Trauma–Grading system for pancreatic trauma.

Grade I	Hematoma. Minor contusion without duct injury. Laceration. Superficial laceration without duct injury.

Grade II	Hematoma. Major contusion without duct injury or tissue loss. Laceration. Major laceration without duct injury or tissue loss.

Grade III	Laceration. Distal transection or parenchymal injury with duct injury.

Grade IV	Laceration. Proximal transection or parenchymal injury with duct injury

Grade V	Laceration. Massive destruction of the pancreatic head

**Table 2:** Review of laparoscopic management for pancreatic trauma in adult patients.

	Cases and gender	Age (years)	Presenting symptoms and amylase/lipase (U/L)	Grade of pancreatic trauma	Associated injuries	Time lapse for surgery (hours)	Laparoscopic management	Operative time (minutes)	Blood loss (ml)	Post-operative stay (days)	Morbidity Clavien I-II III-IV	Type of morbidity	Mortality	Readmission within 30 days	Main outcome
Chin et al. [9]	1 F	22	Epigastric pain with tenderness 592/373	III	No	72	DPS	122	400	5	No	—	No	No	na

Vijay et al. [11]	1 M	28	Epigastric pain with tenderness 108/404	II	Grade I liver lacerations	Conservative management at the beginning	LD	na	na	36	I-II	Pancreatic fistula and collection	No	No	No collection at 6 months

Dardenne [12]	1 M	24	Epigastric pain with tenderness na/337	III	Grade I liver hematoma	24	SPDP followed 1 day later by intramuscular islet autotransplantation	na	na	9	No	—	No	No	na

Pujahari [13]	1 F	32	Upper abdominal pain 1260/na	IV	No	72	LD with jejunostomy	na	na	4	I-II	Pancreatic fistula (grade B)	No	No	Asymptomatic stricture of the pancreatic duct at 3 years

Li et al. [14]	1 F	33	na 865/na	III	No	6	SPDP	na	500	21	I-II	Pancreatic collection	No	No	No collection at 4 years

De Wilt et al. [15]	1 F	34	Upper abdominal pain 52/na	III	Duodenal hematoma	na	SPDP	180	na	10	No	—	No	No	na

Reynolds et al. [16]	1 M	18	Severe abdominal pain and vomiting 850/246	III	No	12	SPDP	na	500	7	No	—	No	No	No collection at 8 weeks

Wolf et al. [17]	1 F	24	Increasing abdominal pain Amylase ↑ ↑	IV	Grade I liver lacerations	48	Endoscopic stent and LD	na	na	14	I-II	Pancreatic collection	No	No	No collection at 1 year

Kalimi et al. [18]	1 F	33	Deteriorating epigastric pain with tenderness 162/na	II	No	na	LD	na	na	44	No	—	No	No	No collection at 4 years

Sayad et al. [19]	1 F	34	Stab wound to the lower back na/na	na	No	na	LD	na	na	8	No	—	No	na	na

Our case	1 M	18	Epigastric pain with tenderness, vomiting 986/156	III	Grade I liver lacerations	Conservative management at the beginning 144	LSR	180	200	6	I-II	Pancreatic fistula (grade B)	No	No	No collection at 24 months

DPS: distal pancreatic splenectomy; SPDP: spleen-preserving distal pancreatectomy; LSR: laparoscopic suture repair; LD: laparoscopic drainage.

**Table 3:** Review about laparoscopic management of pancreatic trauma in paediatric patients.

	Cases and gender	Age (years)	Presenting symptoms and amylase/lipase (U/L)	Grade of pancreatic trauma	Associated injuries	Time lapse for surgery (hours)	Laparoscopic management	Operative time (minutes)	Blood loos (ml)	Post-operative stay (days)	Morbidity Clavien I-II III-IV	Type of morbidity	Mortality	Re-admission within 30 days	Main outcome
Nachiappan et al. 2022 [20]	1F	15	Epigastric pain with tenderness	III	No	24	SPDP	Na	Na	6	No	—	No	No	8 years

Appukuttan et al. 2022 [21]	1 M	17	Na	II	Grade II duodenal injury with duodenal hematoma and grade II hepatic injury	Conservative management until 168168	LD with gastro-jejunostomy	Na	Na	20	IIIb (emergency open Whipple procedure)	Duodenal bleed, luminal bleed	No	No	No

Kanack et al. 2018 [22]	1 M	5	Na	III	No	In emergency	SPDP	Na	Na	9	No	—	No	Collection underwent percutaneous drainage	No

Marom 2018 [23]	1F	15	Epigastric pain	III	No	48	SPDP	Na	Na	Na	No	—	No	No	Na

Gadiyaram et al. 2017 [24]	1 F	15	Abdominal tenderness	IV	No	24	SPDP	Na	Na	7	No	—	No	No	Na

De Vos et al. 2013 [25]	1 M 1 M	12 3	Na	III III	No splenic hematoma	In emergency	SPDP SPDP	Na Na	Na Na	5 7	No No	- -	No No	No No	No collection at 6 weeks

Iqbal et al. 2012 [26]	4 M/3 F	Mean: 8.6	Na	III	No	Mean: 24	SPDP	Mean: 218	Na	Mean: 9.6	4/7 Clavien II	Pancreatic leak, postoperative ileus, wound infection	No	1/7	Na

Rutkoski et al. 2011 [27]	1 F 1 M 1 M	8 10 13	Na 667/533 1783/8399 244/2293	III III III	No No Grade II splenic laceration + left pleural effusion	23 48 72	SPDP SPDP SPDP	344 150 146	100 100 50	6 15 7	No I-II I-II	1 case: Mild pancreatitis 1 case: Abdominal wall hematoma	No No No	No No No	No collection at 12 months 35 months 34 months

Malek et al. 2010 [28]	1 M	13	Abdominal pain amylase and lipase ↑ ↑	III	Grade II splenic laceration	72	SPDP	Na	Na	7	No	**—**	No	No	Na

Nikfarjam et al. 2009 [29]	1 M	14	Epigastric pain with tenderness and vomiting Na/3600	IV	No	Na	DPS	No	50	9	I-II	Pancreatic fistula grade A	No	No	No collection at 1 month

Sayad et al. 2001 [19]	1 M	10	Na	III	Na	Na	SPDP	Na	Na	3	No	—	No	No	Na

DPS: Distal pancreatic-splenectomy; SPDP: Spleen preserving distal pancreatectomy; LSR: Laparoscopic suture repair; LD: Laparoscopic drainage.

## Data Availability

The data used to support the findings of this study are available from the corresponding author upon request.

## Supplementary Materials

Supplementary Materials Video clip surgical procedure—laparoscopic management of pancreatic trauma.
