# Supplementary material for: Laparoscopic Management of Blunt Pancreatic Trauma in Adults and Pediatric Patients: A Systematic Review
Source: Biomed Res Int. 2023 Sep 28;2023:9296570. doi: 10.1155/2023/9296570 (PMC10555496; doi:10.1155/2023/9296570)
Supplement: Supplementary Materials — Video clip surgical procedure—laparoscopic management of pancreatic trauma. [file 9296570.f1.docx]

| Download link [https://wetransfer.com/downloads/72c9d2c6c3fcb63c2add75067ee8ff0620230430153429/e591a0c4b012eb0024a53a17d3239d1a20230430153451/e72be0](https://urldefense.com/v3/__https:/wetransfer.com/downloads/72c9d2c6c3fcb63c2add75067ee8ff0620230430153429/e591a0c4b012eb0024a53a17d3239d1a20230430153451/e72be0?trk=TRN_TDL_01&utm_campaign=TRN_TDL_01&utm_medium=email&utm_source=sendgrid__;!!N11eV2iwtfs!oIyd_hGLOKxWLYk9D99m0Ifm7NqF81ZjCYls9sOJ7CUJvi4CeV5f5FCl2y5gRkbWgsktgSeuf_dj-3AzMnZpvp5tHL7f$) |
| --- |
| 1 item |
